# The effect of unilateral inguinal hernia surgery performed at different times of the day on inflammation and recovery: a prospective randomized controlled study

**DOI:** 10.1007/s13304-026-02669-9

**Published:** 2026-05-19

**Authors:** Burcu Dikmen Demiryorgan, Burak Nalbant, Feyyaz Demiryorgan, Fatma Kavak Akelma

**Affiliations:** 1 Department of Anesthesiology and Reanimation, Ankara Bilkent City Hospital, Ankara, Turkey; 2 Department of Anesthesiology and Reanimation, Faculty of Medicine, Ankara Yildirim Beyazit University, Universities district, 06800 Ankara, Turkey; 3 Department of General Surgery, Ankara Bilkent City Hospital, Ankara, Turkey

**Keywords:** Circadian rhythm, Inflammation, Postoperative sleep quality, Surgical timing, QoR-15 score

## Abstract

**Background:**

Inguinal hernia surgery may trigger inflammation, and circadian variations in surgical timing could affect this response along with postoperative recovery. This study investigated how surgery timing influences inflammation, sleep quality, recovery, and pain in patients undergoing hernia repair.

**Method:**

This prospective, randomized controlled trial was conducted at Ankara Bilkent City Hospital, with 70 patients undergoing elective unilateral inguinal hernia repair performed with a standardized open surgical technique. Participants, aged 18–70 and classified as ASA (American Society of Anesthesiologists) I–II, were randomized into two groups based on surgery timing: morning group (08:00–12:00, Group D) and evening group (18:00–22:00, Group N). Both groups followed standardized institutional fasting protocols, resulting in comparable fasting durations prior to surgery. All patients completed the Athens Insomnia Scale (AIS) preoperatively, and blood samples were collected to measure inflammatory markers including Neutrophil-to-Lymphocyte Ratio (NLR), Platelet-to-Lymphocyte Ratio (PLR), Mean Platelet Volume (MPV), Systemic Inflammation Index (SII), C-reactive Protein (CRP), fibrinogen, Erythrocyte sedimentation rate (ESR), procalcitonin (PCT), ferritin, and Interleukin-6 (IL-6). These parameters were reassessed 24 h after surgery, and AIS was re-evaluated again on postoperative day 3.

**Results:**

Sixty patients were included in the final analysis, with comparable demographics across groups. All inflammatory markers except MPV increased postoperatively in both groups; however, only IL-6 showed a significant between-group difference, with higher levels observed in the night group (*p* < 0.001). Sleep quality was better in the day group on postoperative days 1 and 3 (*p* = 0.05; *p* = 0.019), and recovery scores were also higher (*p* = 0.001). Pain scores were similar except at the 8th hour, where the day group reported lower NRS (Numerical Rating Scale) scores (*p* = 0.032).

**Conclusion:**

Daytime surgeries were associated with lower postoperative IL-6 levels and improved postoperative sleep quality and recovery. Although a statistically significant difference in pain scores was observed at the 8th postoperative hour, this finding was modest and did not affect overall analgesic consumption. These results suggest that surgical timing may influence certain short-term postoperative outcomes. These findings are specific to unilateral open inguinal hernia repair and may not be generalizable to more extensive, complex, or emergency surgical procedures.

**Trial Registration:**

ClinicalTrials.gov (Registration No: NCT06345313, Date: 2024-03-27).

## Introduction

Unilateral inguinal hernia repair is a commonly performed elective surgical procedure worldwide, primarily aimed at preventing complications such as bowel obstruction and strangulation [1]. Surgical trauma is associated with a systemic inflammatory response, typically characterized by elevated levels of C-reactive protein (CRP), interleukin-6 (IL-6), leukocytes, and neutrophils [2, 3]. Inguinal hernia repair has been shown to amplify this inflammatory response [4]. Cytokines play a central role in regulating the immune response to infection, inflammation, and tissue injury, with pro-inflammatory cytokines contributing to the progression and intensity of inflammation. The postoperative increase in cortisol and pro-inflammatory cytokines highlights the close interaction between the physiological stress response and inflammation [5, 6]. Notably, the timing of surgery may affect inflammatory parameters, as cortisol secretion follows a circadian rhythm, potentially influencing both the stress response and postoperative recovery [7].

Surgery and anesthesia can lead to both physical and psychological stress [8]. Among the most common postoperative effects are sleep disturbances and pain, which significantly influence early discharge, morbidity, and overall quality of life. Sleep is regulated by a combination of social, environmental, and physiological factors, with the circadian rhythm—often referred to as the body’s internal clock—playing a key role in controlling sleep, body temperature, and hormone secretion [9]. Various perioperative factors, such as surgical stress, anesthesia methods, and the hospital environment, may negatively affect sleep quality [10].

Postoperative recovery is affected by anesthesia, sleep disturbances, and pain. These perioperative factors play significant roles in the process. Recovery quality is commonly assessed using indicators such as postoperative nausea, early mobilization, and length of hospital stay [11]. The QoR-15 (Quality of Recovery-15) scale offers a patient-centered evaluation by incorporating dimensions such as sleep quality, emotional well-being, physical independence, and pain levels [12].

The primary aim of this study was to evaluate the inflammatory response following unilateral inguinal herniorrhaphy performed at different times of the day. The secondary aim was to compare the effects of surgical timing on postoperative sleep quality, recovery quality, and pain score.

## Materials and methods

### Study design and ethical approval

This prospective, randomized, controlled, single-center trial was conducted at Ankara Bilkent City Hospital, Department of Anesthesiology and Reanimation, Ministry of Health of the Republic of Türkiye, between April 2024 and September 2024. The study received approval from the institutional ethics committee (Approval No: E2-23-4290, dated June 7, 2023) and was registered on ClinicalTrials.gov (Registration No: NCT06345313) on September 16, 2024. All participants provided written and verbal informed consent in accordance with the principles of the Declaration of Helsinki.

## Study population

Eligible participants were patients aged 18 to 70 years, of either sex, classified as ASA (American Society of Anesthesiologists) physical status I or II, literate, Turkish-speaking, and scheduled to undergo elective unilateral inguinal hernia repair performed with a standardized open surgical technique under general anesthesia. Exclusion criteria included refusal to participate, an Athens Insomnia Scale (AIS) score ≥ 6, current hormone or steroid therapy, anti-inflammatory treatment, contraindications to anesthetic drugs, significant visual or auditory impairment, dementia, a BMI (Body Mass Index) > 30, bilateral or complicated inguinal hernia, pregnancy, or the occurrence of unexpected intraoperative surgical complications.

## Randomization and group allocation

Eligible patients were randomized using a computerized system into two groups based on the time of surgery:

- Group D (*n* = 35): surgeries performed between 08:00 and 12:00.
- Group N (*n* = 35): surgeries performed between 18:00 and 22:00.

## Preoperative management

**Fig. 1:**
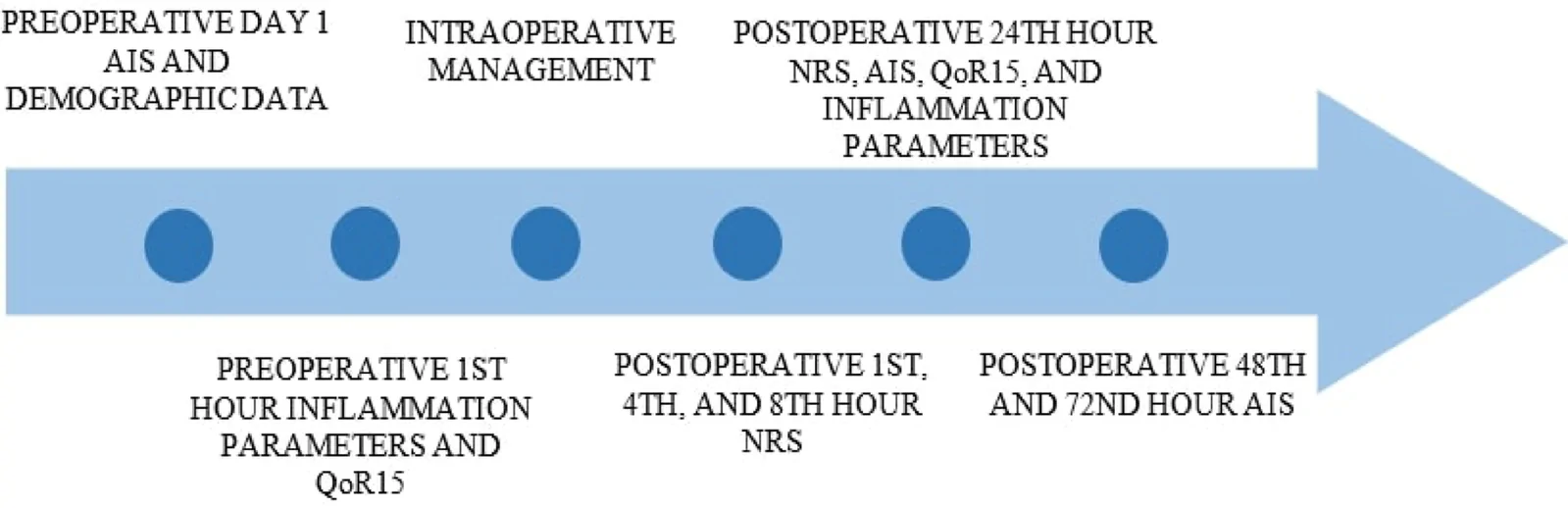
Timeline

All patients fasted for 8 h preoperatively. Group D began fasting after midnight. (00:00 AM), while Group N fasted from 10:00 AM. Although the initiation time of preoperative fasting differed between groups due to surgical scheduling, the total fasting duration was standardized to 8 h in all patients. On the day before surgery, patients completed the Athens Insomnia Scale (0–24 points) to assess sleep quality. One hour prior to surgery, blood samples were collected for preoperative inflammatory markers including Neutrophil-to-Lymphocyte Ratio (NLR), Platelet-to-Lymphocyte Ratio (PLR), Mean Platelet Volume (MPV)(fL), Systemic Inflammation Index (SII)(Neutrophil × Platelet / Lymphocyte), C Reaktif Protein (CRP) (mg/dL), fibrinogen (mg), Erythrocyte sedimentation rate (ESR) (mm/hour), procalcitonin (ng/mL), ferritin (ng/mL), and IL-6 (pg/mL). The Quality of Recovery-15 (QoR-15, 0–150 points) questionnaire was also administered preoperatively (Fig. 1). Demographic data, including age, sex, height, weight, comorbidities, current medications, previous surgeries, and anesthesia history, were recorded.

## Intraoperative management

Patients in Group D underwent surgery between 08:00 and 12:00, and those in Group N between 18:00 and 22:00. Standard monitoring included ECG (Electrocardiogram), NIBP (Non-invasive blood pressure), SpO₂ (Oxygen saturation), and BIS (Bispectral index) monitoring. Intravenous access was established using a 20 G cannula. Anesthesia induction was achieved with 1 mg/kg lidocaine, 2–3 mg/kg propofol, and 1–2 µg/kg fentanyl. A laryngeal mask airway was inserted, and mechanical ventilation initiated.

Anesthesia was maintained with sevoflurane (1–1.5% end-tidal concentration) in a 50% air/50% oxygen mixture, along with a continuous remifentanil infusion. Remifentanil was titrated between 0.05 and 0.2 µg/kg/min to maintain BIS values between 40 and 60. Hypotension, defined as a > 20% decrease in MAP (Mean arterial pressure) from baseline, was managed with 200 mL of crystalloid infusion, followed by 5 mg IV (Intravenosus) ephedrine if needed. Bradycardia (Heart rate (HR) < 45 bpm) was treated with 0.1 mg/kg IV atropine. Intraoperative MAP, HR, SpO₂, and BIS values were recorded.

Fifteen minutes before skin closure, 1 mg/kg IV tramadol and 10 mg/kg IV paracetamol were administered for postoperative analgesia. Additionally, 4 mg of IV ondansetron was given for nausea prophylaxis. No further medications were administered during emergence. All procedures were performed by the same experienced surgeon using a standardized open Lichtenstein technique, in which a polypropylene mesh was placed for tension-free repair.

### Postoperative evaluation

All patients were monitored in the post-anesthesia care unit (PACU) for at least one hour. At the first postoperative hour (T1), Numerical Rating Scale (NRS) pain scores and Aldrete scores were recorded (Fig. 1). Patients with an NRS ≥ 4 received an additional 50 mg IV tramadol. Patients with an Aldrete score ≥ 9 were transferred to the surgical ward.

Postoperatively, patients received oral paracetamol 1 gram every 8 h. NRS scores were reassessed at 4, 8, and 24 h. For NRS ≥ 4, 1 mg/kg IV tramadol was administered as rescue analgesia. At 24 h postoperatively, the AIS and QoR-15 questionnaires were re-administered. Inflammatory markers were reassessed with the same panel used preoperatively (Fig. 1). All of these scales were administered by a blinded investigator.

### Statistical analysis

Sample size was calculated using G*Power 3.1.9.2 software. Based on a pilot study, assuming a clinically significant difference of 7.8 pg/mL in IL-6 levels at 24 h postoperatively (effect size d = 0.74), a power of 80%, and α = 0.05, a minimum of 30 patients per group was required. Accounting for a 15% dropout rate, a total of 70 patients were included.

Descriptive statistics for continuous variables are reported as mean ± standard deviation or median (interquartile range), and categorical variables as frequencies and percentages. The Kolmogorov-Smirnov test was used to assess normality. Pre- and postoperative values were compared using the Wilcoxon signed-rank test. Changes in AIS over time were analyzed with Friedman’s ANOVA, and post-hoc analysis was performed with Friedman’s multiple comparison test. Between-group comparisons were conducted using the independent samples t-test for normally distributed variables and the Mann–Whitney U test for non-normal distributions. Categorical variables were analyzed using the Chi-square or Fisher’s exact test as appropriate. All statistical analyses were performed using IBM SPSS (SPSS – Statistical Package for the Social Sciences) statistics for Windows, Version 20.0 (SPSS Inc., Chicago, IL), with a significance level set at *p* < 0.05.

## Results

**Fig. 2:**
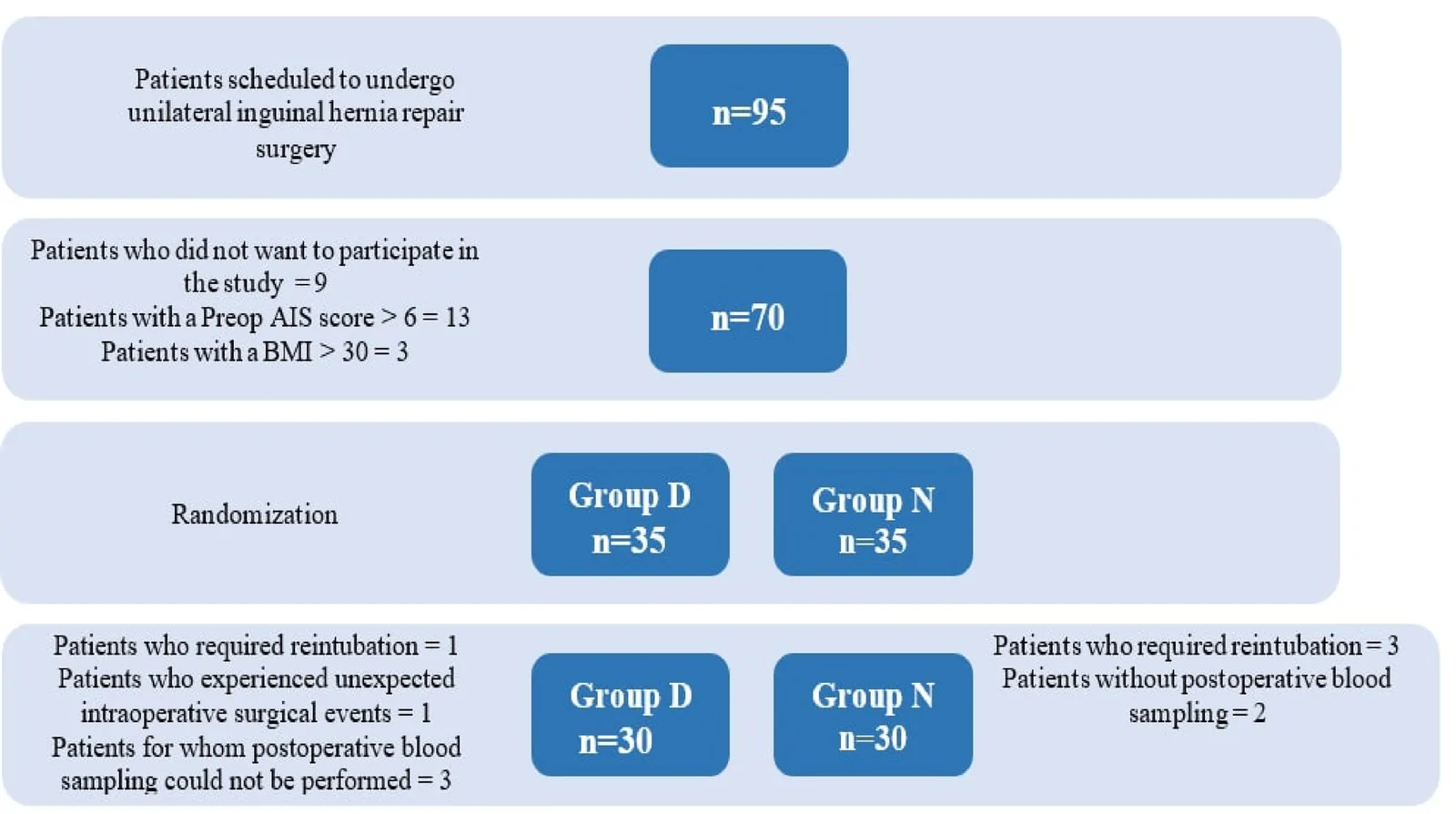
Flow diagram

A total of 95 patients scheduled for unilateral inguinal hernia surgery were assessed for eligibility and underwent preoperative anesthesia evaluations. Of these, 9 patients declined to participate, 13 patients had an AIS score of 6 or higher, and 3 patients had a BMI of 30 or above, thus failing to meet the inclusion criteria. Consequently, 20 patients were excluded, and the study commenced with 70 participants, evenly randomized into two groups (35 in the Day group [Group D] and 35 in the Night group [Group N]). During the course of the study, airway management was converted from laryngeal mask to endotracheal intubation in 4 patients, postoperative blood samples could not be collected from 5 patients, and 1 patient developed an unexpected intraoperative surgical complication. As a result, an additional 10 patients were excluded from the analysis, which was ultimately conducted with 60 patients (*n* = 30 per group) (Fig. 2).

Demographic and clinical characteristics, including age (*p* = 0.238), BMI (*p* = 0.581), duration of surgery (*p* = 0.089), gender distribution (*p* = 1.00), presence of comorbidities (*p* = 0.39) and medication history (*p* = 0.76), were comparable between the two groups (*p* > 0.05) (Table 1). Intraoperative anesthetic characteristics were comparable between the groups. The duration of anesthesia was similar in the day and night groups (67.8 ± 20.3 vs. 60.7 ± 24.9 min, respectively; *p* = 0.227). Likewise, total sevoflurane consumption (29.0 ± 8.1 vs. 25.8 ± 9.3 mL; *p* = 0.161) and total remifentanil dose (0.55 ± 0.19 vs. 0.55 ± 0.22 mg; *p* = 0.975) did not differ significantly between the groups.

Across both groups, postoperative levels of NLR, PLR, SII, CRP, fibrinogen, ESR, procalcitonin, ferritin, and IL-6 were significantly elevated compared to preoperative levels (*p* < 0.05), while MPV levels remained unchanged (*p* > 0.05). There were no significant differences between the groups in the preoperative and postoperative period levels of NLR, PLR, MPV, SII, CRP, fibrinogen, sedimentation rate, procalcitonin, and ferritin (*p* > 0.05). Although preoperative IL-6 levels were similar between the groups (*p* = 0.564), postoperative IL-6 levels were higher in Group N compared to Group D (*p* < 0.001) (Table 2).

Preoperative AIS scores were also similar between the groups (*p* > 0.05). However, a significant increase in AIS scores was observed from the preoperative period to both postoperative day 1 and day 3 in all patients (*p* < 0.05). In Group D, AIS scores significantly differed between postoperative day 1 and day 3 (*p* = 0.035), whereas no such difference was found in Group N (*p* = 0.244). Furthermore, AIS scores on both postoperative day 1 and day 3 were significantly higher in Group N compared to Group D (*p* = 0.05 and *p* = 0.019, respectively) (Tabel 3).

Preoperative QoR-15 scores did not differ significantly between the groups (*p* > 0.05). However, a significant decline in QoR-15 scores was observed postoperatively in all patients (*p* < 0.001). Postoperative QoR-15 scores were significantly lower in Group N compared to Group D (*p* = 0.001). Regarding NRS pain scores, no significant differences were noted between the groups at the 1st (*p* = 0.323), 4th (*p* = 0.517), and 24th hours (*p* = 0.192) but group N exhibited significantly higher pain levels at the 8th hour (*p* = 0.032) (Table 3).

**Table 1:** Demographic data

	Day group (*n* = 30)	Night group (*n* = 30 )	*p*-value
Age (n)	46.03 ± 14.65	49.96 ± 10.53	0.238^a^
BMI (kg/m^2^) (n)	25.67 ± 2.94	26.10 ± 3.00	0.581^a^
Operation time (min)	67.83 ± 20.32	60.66 ± 24.86	0.089^b^
Gender (n) (%)			1.000^c^
Male	28 (93.3)	28 (93.3)	
Female	2 (6.7)	2 (6.7)	
*Comorbidities (n) (%)*			0.390^c^
Yes	7 (23.3)	10 (33.3)	
Hypothyroidism	2 (6.7)	3 (10.0)	
Hypertansion	2 (6.7)	0 (0)	
Diabetes Mellitus	0 (0)	2 (6.7)	
Arrhythmia	2 (6.7)	0 (0)	
BPH	0 (0)	1 (3.3)	
Prostate Cancer	0 (0)	1 (3.3)	
Multiple Sclerosis	1 (3.3)	1 (3.3)	
Hyperlipidemia	0 (0)	1 (3.3)	
Asthma	0 (0)	1 (3.3)	
*Drugs used (n) (%)*			0.766^c^
Yes	7 (23.3)	8 (26.7)	
Levothyroxine	2 (6.7)	3 (10)	
Antihypertensive	2 (6.7)	0 (0)	
Oral Antidiabetic	0 (0)	2 (6.7)	
Beta Blocker	2 (6.7)	0 (0)	
Tamsulosin Hcl	0 (0)	1 (3.3)	
SSRI	1 (3.3)	1 (3.3)	
SNRI	0 (0)	1 (3.3)	

a: Independent Samples t test, b:Mann Whitney U test, c: Chi -Square test/Fisher’s Exact Test

**Table 2:** Inflammatory markers at baseline and after surgery

	Day Group (D) (*n* = 30)	Night Group (*N*) (*n* = 30)	*p*-value*
NLR Preoperative Postoperative *p*-value**	2.23 ± 0.81 4.75 ± 1.71 **< 0.001**	2.16 ± 0.72 5.74 ± 0.07 **< 0.001**	0.894 0.315
PLR Preoperative Postoperative p-value**	125.82 ± 39.42 151.84 ± 67.43 **0.019**	110.06 ± 32.10 153.95 ± 86.56 **< 0.001**	0.090 0.469
MPV Preoperative Postoperative p-value**	7.94 ± 1.02 7.70 ± 0.78 0.466	7.86 ± 0.57 8.00 ± 0.64 0.470	0.620 0.125
SII Preoperative Postoperative p-value**	550.59 ± 263.69 1080.66 ± 444.35 **< 0.001**	514.083 ± 255.03 1155.32 ± 628.38 **< 0.001**	0.647 0.712
CRP Preoperative Postoperative p-value**	3.12 ± 4.18 29.04 ± 17.84 **< 0.001**	4.85 ± 6.60 31.59 ± 16.96 **< 0.001**	0.207 0.337
Fibrinogen Preoperative Postoperative p-value**	2.67 ± 0.54 3.19 ± 0.56 **< 0.001**	1.77 ± 0.71 3.29 ± 0.51 **< 0.001**	0.739 0.351
Sedimentation Preoperative Postoperative p-value**	5.80 ± 2.74 9.20 ± 5.32 **< 0.001**	7.63 ± 7.11 10.56 ± 8.84 **0.026**	0.970 0.778
Procalcitonin Preoperative Postoperative p-value**	0.03 ± 0.01 0.06 ± 0.06 **0.004**	0.04 ± 0.08 0.05 ± 0.08 **0.015**	0.214 0.647
Ferritin Preoperative Postoperative p-value**	75.66 ± 67.87 88.40 ± 60.86 **< 0.001**	67.80 ± 80.67 87.16 ± 92.05 **< 0.001**	0.525 0.657
IL-6 Preoperative Postoperative p-value**	3.24 ± 1.96 11.52 ± 6.37 **< 0.001**	2.90 ± 0.53 26.64 ± 14.64 **< 0.001**	0.564 **< 0.001**

*: Wilcoxon test, **: Mann Whitney U test, Values in bold type are statistically significant (*p*<0.05)

CRP: C-Reactive Protein; IL-6: Interleukin-6; MPV: Mean Platelet Volume; NLR: Neutrophil-to-Lymphocyte Ratio; PLR: Platelet-to-Lymphocyte Ratio; SII: Systemic Inflammation Index.

**Table 3:** AIS, QoR15 and NRS at baseline and after surgery

	Day group (D) (*n* = 30)	Night group (*N*) (*n* = 30)	*p*-value*
AIS Preoperative Postoperative 1 Postoperative 3 p-value**	2 (0–5) 3 (1–8) 2.5 (0–7) **< 0.001**	2 (0–4) 4 (2–9) 3.5 (1–9) **< 0.001**	0.892 **0.05** **0.019**
QoR 15 Preoperative Postoperative p-value***	148 (145–150) 131 (111–140) **< 0.001**	147 (140–150) 126.5 (117–134) **< 0.001**	0.126 **0.001**
NRS 1 4 8 24	5 (3–9) 5 (3–8) 4 (2–7) 3 (1–7)	5 (2–7) 5 (3–6) 5 (3–7) 4 (1–7)	0.323 0.517 **0.032** 0.192

*: Mann Whitney U test, **: Friedman Test, ***: Wilcoxon test, Values in bold type are statistically significant (*p*<0.05)

AIS: Athens Insomnia Scale, QoR 15: Quality of Recovery 15

## Discussion

The timing of surgery appears to play an important role in postoperative inflammation, sleep quality, recovery, and pain. In this study, we evaluated the impact of performing unilateral inguinal hernia repairs during the daytime versus at night, using a standardized open Lichtenstein technique; therefore, the findings should not be generalized to laparoscopic procedures or to bilateral or complex hernia repairs. Both groups adhered to institutional fasting protocols that resulted in comparable preoperative fasting durations. Although Group D fasted after midnight and Group N began fasting at 10:00 AM, alignment with their respective surgery times yielded equivalent total fasting intervals,. making it less likely that differences in fasting duration contributed to postoperative outcomes; however, the potential influence of circadian timing of fasting initiation cannot be completely excluded. Although fasting duration was standardized, the timing of fasting initiation differed between groups. Circadian timing of metabolic stressors such as fasting may influence inflammatory responses; however, direct clinical evidence on its effect in postoperative settings remains limited. Inflammation increased in both groups after surgery; however, a significant between-group difference was observed only for IL-6, which was higher in patients who underwent surgery at night. Additionally, daytime procedures were associated with better postoperative sleep quality and improved recovery scores, suggesting that operations performed earlier in the day may lead to more favorable short-term clinical outcomes.

Postoperative inflammation is a widely recognized response to surgical procedures [3]. In the present study, elevated levels of all inflammatory markers were observed in the postoperative period, irrespective of the timing of surgery. Roch et al. highlighted IL-6 as a key biomarker for postoperative inflammation in spinal surgeries, noting its important role in the early detection of infections [13].

A substantial body of research has evaluated inflammation markers and the circadian rhythm at the molecular level. Research investigating the clinical responses related to the circadian rhythm has generally focused on the daily rhythm of cortisol. In their molecular-level study, Keller et al. examined the effects of the circadian rhythm on IL-6, TNF-α (Tumor Necrosis Factor-alpha), macrophages, and other inflammatory cytokines, and found that inflammation increased during the dark phase of the day [14]. This finding suggests that the process may be independent of the circadian rhythm of cortisol and may instead reflect an intrinsic circadian rhythm within each lymphoid tissue. In a study conducted by Zeng et al. in [15], it was reported that the cellular origin of proinflammatory cytokine activity remains unclear; however, monocytes are considered the main contributors in peripheral blood. Receptor-mediated lipopolysaccharide ligation was shown to significantly increase the production of IL-6 and TNF-α in peripheral blood monocytes during the dark phase and following sleep deprivation [15]. Based on these molecular-level findings, our clinical study investigated the potential impact of surgeries performed at different times of the day on postoperative inflammation. We found significantly higher IL-6 levels in the group undergoing surgery at night compared to those who had surgery during the day.

Ramji et al., in a study evaluating surgical timing in patients with hip fractures, reported that evening surgeries heightened the inflammatory response, leading to prolonged postoperative complications and extended hospital stay (16). In contrast, Kwon et al. found that afternoon surgeries were associated with a quicker normalization of inflammatory markers, although patients experienced higher levels of pain. This was attributed to diurnal variations in cortisol secretion [7]. Consistent with prior evidence, IL-6 emerged as the most sensitive biomarker of postoperative inflammatory activation, and in our study, it was the only marker that demonstrated a significant between-group difference. IL-6 is an early-response cytokine that rises rapidly within hours of tissue injury and exhibits pronounced circadian sensitivity, which may explain its heightened responsiveness to the timing of surgical stress. In contrast, downstream acute phase reactants such as NLR, PLR, CRP, and fibrinogen display slower kinetics, longer half-lives, and greater dependence on hepatic synthesis and broader regulatory pathways. These features may attenuate or obscure subtle circadian effects at the measured time points, even though postoperative increases were observed across markers. Accordingly, the selective between-group difference in IL-6 may reflect its role as an immediate, circadian-regulated mediator of surgical inflammation and suggests that circadian modulation of postoperative inflammatory responses may be selective rather than global.

Surgeries performed at night were associated with greater disruptions in postoperative sleep quality, with a higher incidence of sleep disturbances. Hou et al. demonstrated that procedures conducted later in the day significantly reduced sleep duration, sleep efficiency, and overall sleep quality compared to those performed earlier in the day [16]. Similarly, Song et al. highlighted differences in anesthetic drug requirements between day and night surgeries, with night procedures having a more detrimental impact on sleep quality [17]. In our study, standard anesthetic techniques were consistently applied in both groups, minimizing the influence of anesthesia on postoperative sleep outcomes. Consistent with these findings, our results indicated that sleep quality—a critical component of postoperative recovery—was more negatively affected by surgeries conducted at night. Using the Athens Insomnia Scale (AIS), we observed notable reductions in sleep duration, sleep efficiency, and overall sleep quality among patients undergoing operations at night. Furthermore, patients who had surgery during the day experienced a faster recovery in sleep quality by the third postoperative day, likely due to a shorter disruption of their biological and circadian rhythms. Concordantly, overall quality of recovery was superior in the daytime group, as reflected by significantly higher QoR-15 scores, supporting a more favorable early multidimensional postoperative recovery profile.

Postoperative pain is an important determinant of recovery, and night-time surgeries have previously been associated with increased pain perception and higher analgesic requirements, as reported by Cicekci et al. [18]. In our study, pain scores were higher in the night group only at the 8th postoperative hour; however, this difference was modest, restricted to a single time point, and not accompanied by greater analgesic consumption or delayed recovery. Given these characteristics, the clinical relevance of this transient elevation appears limited and should be interpreted cautiously. Circadian variations in early postoperative stress and nociceptive sensitivity—along with the known relationship between postoperative pain and impaired sleep quality—may contribute to this short-lived increase in pain perception, a phenomenon also supported by findings from Song et al., who reported time-of-day differences in anesthetic and analgesic responsiveness [17].

### Limitations

This study has several limitations. First, it focused on a medium-scale surgical procedure—inguinal hernia repair—which may limit the generalizability of the findings to other types of surgeries. Second, the study evaluated only short-term postoperative outcomes and did not investigate the long-term effects of surgical timing on recovery and the development of potential chronic conditions. Third, because IL-6 was the pre-specified primary endpoint, the additional inflammatory biomarkers were analyzed as exploratory outcomes. These secondary analyses were not corrected for multiple comparisons, and their results should therefore be interpreted with caution due to the potential for inflated type I error. In addition, although the total preoperative fasting duration was standardized, differences in the timing of fasting initiation may represent a potential physiological confounder and should be considered when interpreting the results. Finally, although mixed-model approaches may offer advantages for repeated-measures outcomes, the small sample size and non-normal distribution of AIS and QoR-15 scores made non-parametric methods more appropriate in this study. Future research should include a broader range of surgical procedures and assess long-term outcomes to more comprehensively evaluate the impact of surgical timing on postoperative inflammation.

## Conclusion

Surgical planning that takes into account the body’s natural circadian rhythms has emerged as a promising approach to optimizing clinical outcomes and enhancing recovery. Our findings suggest that aligning surgical timing with the body’s natural circadian rhythms may improve postoperative outcomes, particularly through lower postoperative IL-6 levels, enhancing sleep quality, accelerating recovery, and alleviating pain perception. Inguinal hernia repairs performed during the day resulted in significantly lower IL-6 levels and improved recovery profiles compared to night surgeries. These findings apply exclusively to unilateral inguinal hernia repairs performed using an open surgical technique and should not be extrapolated to laparoscopic procedures, locally anesthetized repairs, or more complex hernia types. Given the restricted scope of this study, which focused solely on a single medium-scale elective procedure, the generalizability of these results to larger, complex, or emergency surgeries is limited and should be interpreted with caution. These results underscore the clinical relevance of surgical timing and highlight the need for further studies across diverse surgical procedures to confirm and generalize these findings.

## Data Availability

The datasets generated and analyzed during the current study are available from the corresponding author on reasonable request.
